# The Utility of Thirst as a Measure of Hydration Status Following Exercise-Induced Dehydration

**DOI:** 10.3390/nu11112689

**Published:** 2019-11-07

**Authors:** William M. Adams, Lesley W. Vandermark, Luke N. Belval, Douglas J. Casa

**Affiliations:** 1 Department of Kinesiology, University of North Carolina at Greensboro, 1408 Walker Avenue, 237L Coleman Building, Greensboro, NC 27412, USA; 2 Department of Health, Human Performance, & Recreation, University of Arkansas, HPER 310D, Fayetteville, AR 72701, USA; 3 Institute for Exercise and Environmental Medicine, Texas Health Presbyterian Hospital Dallas and University of Texas Southwestern Medical Center, 7232 Greenville Ave, Dallas, TX 75231, USA; 4 Korey Stringer Institute, Human Performance Laboratory, Department of Kinesiology, University of Connecticut, 2095 Hillside Rd, Unit 1110, Storrs, CT 06269, USA

**Keywords:** fluid replacement, hypohydration, assessment, perception, exercise

## Abstract

The purpose of this study was to examine the perception of thirst as a marker of hydration status following prolonged exercise in the heat. Twelve men (mean ± SD; age, 23 ± 4 y; body mass, 81.4 ± 9.9 kg; height, 182 ± 9 cm; body fat, 14.3% ± 4.7%) completed two 180 min bouts of exercise on a motorized treadmill in a hot environment (35.2 ± 0.6 °C; RH, 30.0 ± 5.4%), followed by a 60 min recovery period. Participants completed a euhydrated (EUH) and hypohydrated (HYPO) trial. During recovery, participants were randomly assigned to either fluid replacement (EUH_FL_ and HYPO_FL_; 10 min ad libitum consumption) or no fluid replacement (EUH_NF_ and HYPO_NF_). Thirst was measured using both a nine-point scale and separate visual analog scales. The percent of body mass loss (%BML) was significantly greater immediately post exercise in HYPO (HYPO_FL_, 3.0% ± 1.2%; HYPO_NF_, 2.6% ± 0.6%) compared to EUH (EUH_FL_, 0.2% ± 0.7%; EUH_NF_, 0.6% ± 0.5%) trials (*p* < 0.001). Following recovery, there were no differences in %BML between HYPO_FL_ and HYPO_NF_ (*p* > 0.05) or between EUH_FL_ and EUH_NF_ (*p* > 0.05). Beginning at minute 5 during the recovery period, thirst perception was significantly greater in HYPO_NF_ than EUH_FL_, EUH_NF_, and HYPO_FL_ (*p* < 0.05). A 10 min, ad libitum consumption of fluid post exercise when hypohydrated (%BML > 2%), negated differences in perception of thirst between euhydrated and hypohydrated trials. These results represent a limitation in the utility of thirst in guiding hydration practices.

## 1. Introduction

The complexities surrounding the turnover of body water complicates any single measure of hydration status qualifying as a standard for assessment [1]. Methods for assessing hydration status utilize urinary and hematologic measures, among others; however, these methods are not without limitations regarding accuracy and applicability in all settings [1,2,3,4]. Furthermore, many of these assessment methods require expensive laboratory instrumentation and/or expertise in these techniques, limiting real-world applicability for all persons.

Thirst, defined as a desire to consume fluids as a result of a body water deficit, is a subjective perception controlled by both neuroendocrine responses to maintain fluid homeostasis [5,6,7] and psychosocial influences [8]. The physiological onset of thirst, occurring with fluid losses of a magnitude of 1–2% body mass loss [6,9] is influenced by hyperosmolality, hormonal responses (arginine vasopressin (AVP) and angiotensin II (Ang II)), and peripheral osmoreceptors [10,11,12], but is highly variable within individuals [5]. In addition, non-homeostatic influences, such as beverage taste, availability, individual drinking habits, and timing with meals, dictate daily fluid intake [8].

Current recommendations advocate for using thirst to guide hydration practices during exercise to reduce the risk of exertional hyponatremia [13]; however, recommendations by the American College of Sports Medicine [14] and the National Athletic Trainers’ Association [15] encourage individualized replacement of fluid losses to prevent dehydration-mediated loss of >2% of body mass during exercise. Evidence suggests that relying solely on thirst as a means of replacing fluid losses during exercise, especially in hot environmental conditions, may prevent the full restoration of body water losses, leading to involuntary dehydration [6]. Armstrong et al. [16] has suggested that thirst may provide an inexpensive means of assessing hydration status upon waking in the morning, but differences in thirst perception were negated following the consumption of a bolus of fluid, despite hypohydration equaling 2% body mass loss. Similar findings were observed when ad libitum consumption of water was allowed following high intensity intermittent exercise; however, body water deficits were only 1.3% of body mass [17].

An assessment of thirst’s tracking of hydration status has previously been performed during exercise and at rest. However, few studies have investigated the efficacy of thirst as a potential marker of hydration status when a bolus of water was provided following exercise-induced dehydration upon a magnitude of ~3% body mass loss. Therefore, the purpose of this study was to examine the perception of thirst as a marker of hydration status following prolonged exercise in the heat. It was hypothesized that when a bolus of fluid was consumed following exercise-induced dehydration, thirst sensation would be attenuated despite a sustained level of dehydration above the threshold (~2% body mass loss from dehydration) in which thirst is present.

## 2. Materials and Methods

### 2.1. Design

Participants completed two testing sessions in a randomized, counter-balanced manner. We randomly assigned participants to testing sessions, designed to manipulate participants’ hydration states following exercise: euhydrated (EUH) and hypohydrated (HYPO). The EUH trial consisted of a euhydrated arrival to the laboratory, followed by minimal fluid losses throughout the duration of exercise dictated by participant’s individual fluid needs. The HYPO trial was designed to achieve a state of hypohydration of roughly 3% body mass loss and was achieved via 14 h fluid restriction prior to arrival to the laboratory and throughout the bout of exercise. Following exercise, participants completed a 60 min bout of recovery in the heat, where they were randomly allocated to either a fluid replacement group or fluid restriction group. All exercise sessions occurred at the same time of day, ±1 h, and were separated by a minimum of 72 h to minimize the circadian variability of the physiological variables of interest and allow for appropriate recovery from the previous sessions, respectively. Exercise and recovery took place in a climate-controlled environmental chamber (Model 200, Minus-Eleven, Inc., Malden, MA, USA) with conditions being: ambient temperature, 35.2 ± 0.6 °C; RH, 30.0% ± 5.4%; wet bulb globe temperature, 26.6 ± 1.1 °C. Additionally, all exercise sessions took place during the winter months in Connecticut, USA to ensure that none of the participants were heat acclimated.

### 2.2. Participants

Twelve recreationally active men between the ages of 18 and 35 volunteered to participate in this study (mean ± SD; age, 23 ± 4 y; body mass, 81.4 ± 9.9 kg; height, 182 ± 9 cm; body fat, 14.3% ± 4.7%). All participants reported exercising a minimum of 4–5 d·wk^−1^ for at least 30 min per session. Participants were excluded if they reported any chronic health problems, such as cardiovascular, metabolic, or respiratory disease; current illness or musculoskeletal injury; or a previous history of exertional heat illness within the last 3 y. Participants provided written and informed consent to participate in this study, which was approved by the University of Connecticut’s Institutional Review Board (protocol number: H15-154) and in accordance with the Declaration of Helsinki.

### 2.3. Procedures

**Familiarization Session.** Prior to the exercise sessions, participants completed a familiarization session for them to become acquainted with the laboratory and testing procedures. The familiarization session was scheduled as close to the scheduled testing session times as possible, to ensure minimal variability in reference hydration values due to circadian rhythm. To ensure participants arrived euhydrated, they were asked to consume 500 mL of water prior to going to bed the night before and upon waking in the morning. Hydration status was measured upon arrival to the laboratory using urine specific gravity (U_SG_ ≤ 1.020) (refractometer, model A300CL; Atago Inc., Tokyo, Japan) and urine color (U_COL_ ≤ 4) via urine color chart [18]. Participants arriving to the laboratory for the EUH trial with a U_SG_ > 1.020 were instructed to consume an additional 500 mL of fluid prior to the start of exercise. Participants arriving to the laboratory for the HYPO trial with a U_SG_ > 1.020 were not provided fluid, as the purpose of the HYPO trial was to induce a state of hypohydration prior to the start of exercise.

Each participant’s height was measured using a standard stadiometer and their body fat was calculated via three-site skinfold measurements of the chest, abdomen, and thigh using calibrated calipers (Lange Skinfold Caliper; Beta Technology Inc., Santa Cruz, CA, USA) [19]. Participants were instructed on the insertion of the rectal thermometer (Model 401, Measurement Specialties, Hampton, VA, USA), which was used for the monitoring of body temperature during the exercise sessions. They were also familiarized to the perceptual scales that they were asked about during the exercise sessions: the thirst perception (TH) and thirst sensation scales (TSS), described below.

Each participant’s sweat rate was measured to determine their fluid needs during the EUH exercise trial. A nude body mass (NBM) was measured to the nearest 0.1 kg using a calibrated scale (Defender 5000, OHAUS, Parsippany, NJ, USA) before entering the environmental chamber. Upon entering the chamber, they stood for 15 min to become equilibrated to the environmental conditions prior to exercise. Exercise consisted of walking on a motorized treadmill at a speed ranging from 5.6 to 6.4 km·h^−1^ at a 2% gradient for a total of 30 min. Participants were instructed to set a speed equivalent to a “fast walk” that they would be able to sustain for up to 3 h. The speed selected during the familiarization session remained the same for both exercise sessions. Following exercise, participants provided another NBM to determine sweat rate by assessing body mass change. Body water losses were used to quantify the prescribed fluid replacement during exercise of the EUH trial; 0.001 kg equaled 1 mL.

**Testing Sessions.** For all testing sessions, participants were instructed to conduct their normal daily routine (e.g., exercise, food and fluid intake), so as to not deviate from their individual norm. Participants were asked to consume an additional 500 mL of water the night prior and the morning of the EUH trial to ensure a state of euhydration. For the HYPO trial, participants were asked to restrict fluid intake (including fluid heavy foods) for 14 h prior to their arrival to the laboratory. Participants were scheduled for the same time of day for each of their trials to minimize any effects of circadian rhythms on physiologic function.

Upon arrival to the laboratory for the EUH and HYPO trials, we obtained the following measures; NBM, U_SG_, and U_COL_. Following a 15 min equilibration in the environmental chamber, pre-exercise (PRE_EX_) measures of rectal temperature (T_REC_), heart rate (HR) (Race Trainer, Timex Group USA, Middlebury, CT, USA), body mass, TH, and TSS were obtained. A blood sample, with participants in a seated position, was also drawn at this time for the assessment of serum osmolality prior to exercise. Participants then began exercise, consisting of six 30 min bouts of exercise, each involving a 25 min walk at the speed at which participants performed the sweat rate assessment test, followed by a 5 min rest. During the 5 min rest period, participants stepped off the treadmill, removed their shoes and t-shirt, toweled off as much as possible and provided a body mass measure to track body mass loss over the course of exercise. Before commencing the next bout of exercise, T_REC_, HR, RPE, and TH were measured. During the EUH trial, participants consumed equal boluses of water during each 25 min exercise bout at a volume matching their calculated sweat rate for that time period. During the HYPO trial, participants were restricted from water throughout exercise.

Following the 3 h bout of exercise, participants stepped off the treadmill, provided body mass, T_REC_, HR, TH, and TSS post-exercise (POST_EX_) measures, and sat in a chair to begin a 60 min period of recovery. During recovery, participants were randomly assigned to one of two recovery conditions; fluid replacement (FL; EUH_FL_ and HYPO_FL_) or no fluid replacement (NF; EUH_NF_ and HYPO_NF_). Participants remained in the same recovery group for both trials. For EUH_FL_ and HYPO_FL_ conditions, participants were given a bolus of water equaling their total body mass losses that occurred during exercise. The participants were allotted 10 min to consume the water and were permitted to consume the water ad libitum. After 10 min, any remaining water was taken from the participants and they remained in a seated position for the next 50 min. Following the completion of the recovery portion of the trial, T_REC_ and HR were measured and TH and TSS were assessed for a post-recovery (POST_REC_) time point. Prior to exiting the environmental chamber, a POST_REC_ blood sample was obtained for assessment of serum osmolality. Participants then exited the environmental chamber and provided final NBM, U_SG_, and U_COL_ measures.

### 2.4. Thirst Assessment

TH was measured using nine-point (1–9) Likert scale that provided verbal anchors of 1, “Not Thirsty at All”; 3, “A Little Thirsty”; 5, “Moderately Thirsty”; 7, “Very Thirsty”; and 9, “Very, Very Thirsty” [20]. Participants were asked, “How thirsty are you right now?” when shown the scale, and they provided a numerical answer based on their perceived feeling of thirst.

The second measure of thirst assessment, TSS, was measured using a 100 mm visual analog scale, for which participants were asked, “How thirsty do you feel right now?” (not at all thirsty–very thirsty); “How pleasant would it be to drink some water right now?” (very unpleasant–very pleasant); “How dry does your mouth feel right now?” (not at all dry–very dry); “How would you describe the taste in your mouth?” (normal–very unpleasant); “How full does your stomach feel right now?” (not at all full–very full); “How sick to your stomach do you feel right now?” (not at all sick–very sick) [21,22]. All six visual analog scales were on one piece of paper and each 100 mm line was anchored using the aforementioned words/phrases in parentheses above. Participants were asked to mark on each line their responses to each question.

### 2.5. Hematological Measures

Five milliliters of blood was drawn from an antecubital vein into a collection tube without additive (BD Vacutainer, Becton Dickinson Company, Franklin Lakes, NJ, USA) and allowed to clot at room temperature. Samples were then centrifuged at 3000 rpm at 4 °C and assessed in triplicate for serum osmolality using the freezing point depression method (Model 3320, Advanced Instruments, Norwood, MA, USA). 

### 2.6. Statistical Analysis

All statistical analyses were performed using SPSS Statistical Software version 21 (IBM Corporation, Armonk, NY, USA). Tests for normality were conducted using the Shapiro–Wilk tests with any non-normally distributed data being analyzed by the appropriate non-parametric tests. All values are presented as means ± standard deviations unless otherwise noted. In addition, comparisons between variables are presented as mean differences (MD) and 95% confidence intervals (95%CI). Effect size (ES) was also calculated using Cohen’s d, for which d = 0.2 was considered a small effect, d = 0.5 was considered a medium effect, and d = 0.8 was considered a large effect. A priori power analysis (G*Power 3.1, Düsseldorf, DE) computing an F test for repeated measures ANOVA with within–between interaction for two groups (NF and FL) across 20 timepoints with an alpha level of 0.05, power of 0.8, and a medium effect size of d = 0.5 yielded a total sample size of 8. A sample size of 12 (*n* = 6 were each assigned to each of NF and FL groups) was used to ensure a power of >0.8. 

Three-way (condition × trial × time) repeated-measures ANOVAs were used to assess differences in dependent variables (TH, TSS, body mass, serum osmolality, HR, and T_REC_) between conditions (FL and NF), trials (EUH and HYPO), across time. With significant three-way interactions, follow-up post hoc testing utilizing appropriate two-way ANOVAs were utilized. Significance was set a priori at *p* < 0.05.

Post hoc power analysis comparing TH (comparison of pooled means during recovery) yielded a power-achieved of β = 0.997, confirming that the sample size selected was appropriate for the analysis. Post hoc power analysis comparing the TSS measures of thirstiness, pleasantness, dryness, taste, fullness, and sickness yielded power measures of β = 0.579, β = 0.338, β = 0.516, β = 0.415, β = 0.453, and β = 0.435, respectively.

## 3. Results

**Figure 1:**
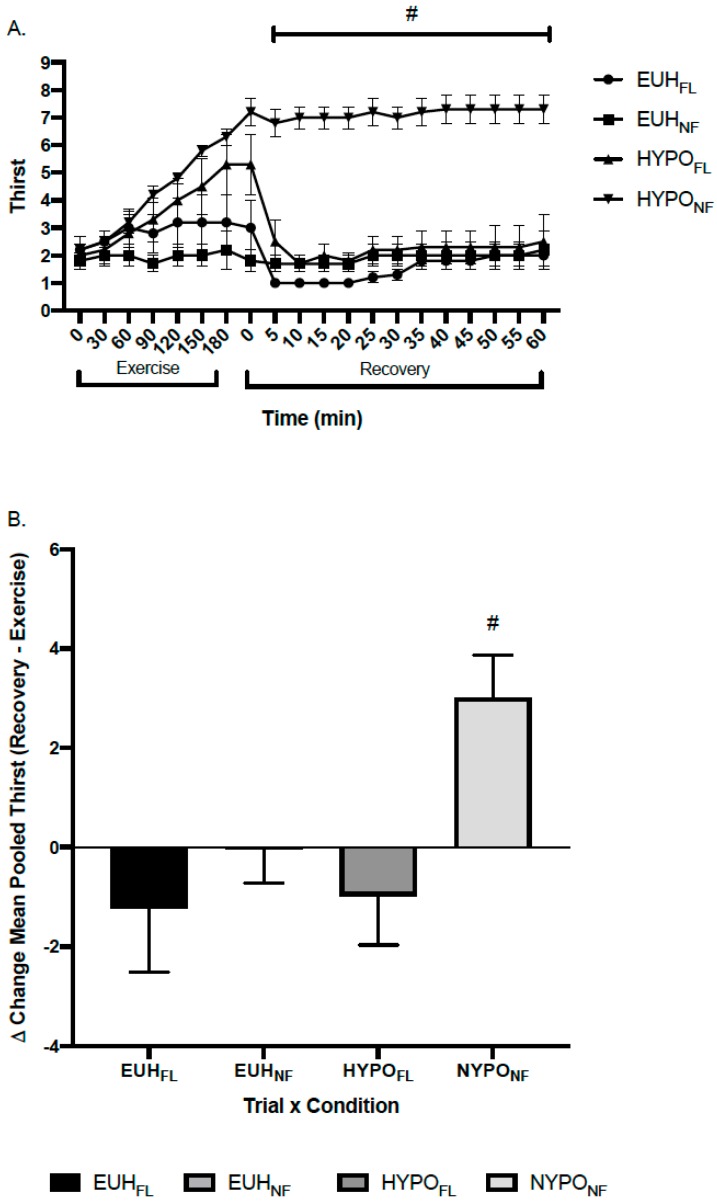
(**A**) Thirst perception throughout exercise and post-exercise recovery and (**B**) delta change (recovery–exercise) of pooled means in thirst perception (TH) by trial × condition. # indicates a significant difference between HYPO_FL_ and HYPO_NF_, EUH_FL_, and EUH_NF_, *p* < 0.05. EUH_FL_ = minimized fluid losses during exercise and remaining losses replaced during recovery; EUH_NF_ = minimized fluid losses during exercise and did not replace losses during recovery; HYPO_FL_ = fluid restricted during exercise and losses replaced during recovery; HYPO_NF_ = fluid restricted during exercise and losses not replaced during recovery.

Figure 1A depicts the change in TH throughout exercise and recovery and Figure 1B depicts the delta change (recovery–exercise) in pooled means for TH. There was a significant three-way interaction for TH between EUH_FL_, EUH_NF_, HYPO_FL_, and HYPO_NF_ (*p* = 0.015). Follow-up testing revealed that, during recovery, mean TH was significantly greater in HYPO_NF_ than EUH_FL_ (*p* < 0.001, ES = 6.44), EUH_NF_ (*p* < 0.001, ES = 5.05), and HYPO_FL_ (*p* = 0.002, ES = 3.70) (Figure 1A).

**Figure 2:**
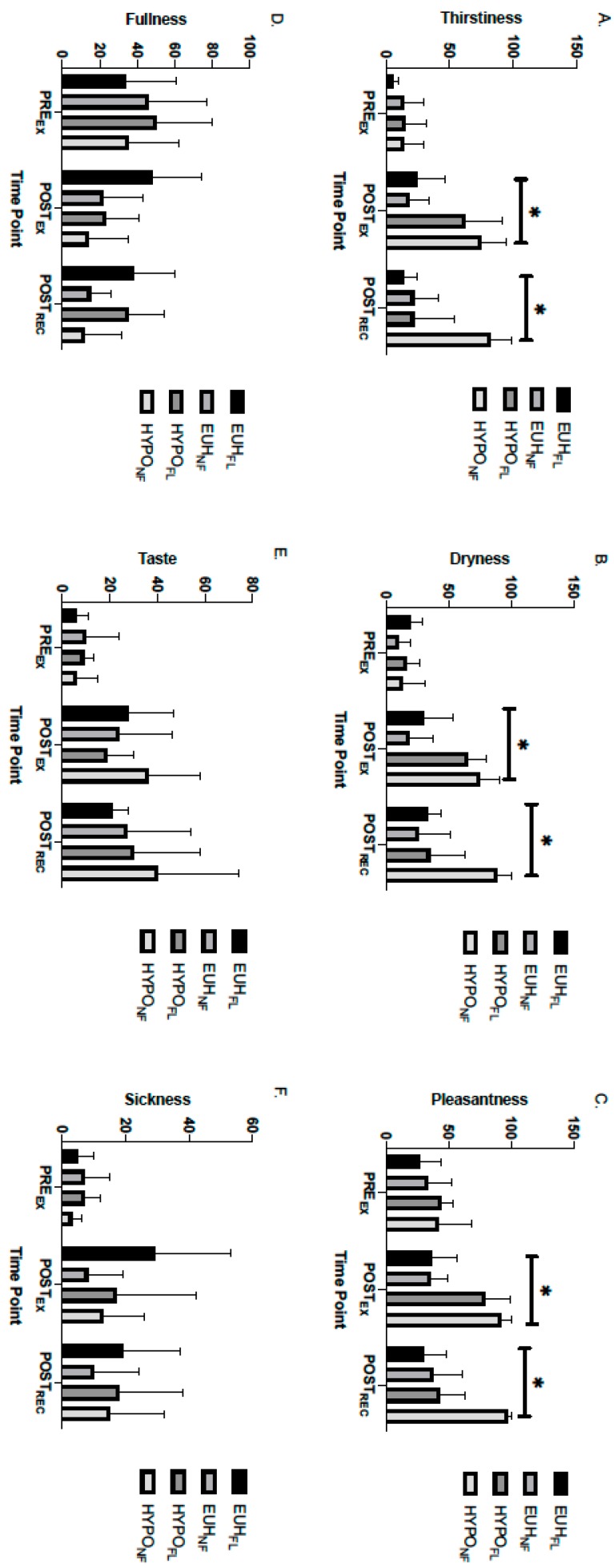
Perceptions of (**A**) thirstiness, (**B**) dryness, (**C**) pleasantness, (**D**) fullness, (**E**) taste, and (**F**) sickness in EUH_FL_ EUH_NF_ HYPO_FL_ and HYPO_NF_ groups. * indicates a significant difference between the HYPO trial (HYPO_FL_ and HYPO_NF_) and EUH trial (EUH_FL_ and EUH_NF_); *p* < 0.05. EUH_FL_ = minimized fluid losses during exercise and remaining losses replaced during recovery; EUH_NF_ = minimized fluid losses during exercise and did not replace losses during recovery; HYPO_FL_ = fluid restricted during exercise and losses replaced during recovery; HYPO_NF_ = fluid restricted during exercise and losses not replaced during recovery.

Figure 2 portrays the separate measures assessed in TSS at the PRE_EX_, POST_EX_, and POST_REC_ time point. There were no significant differences in feelings of thirstiness (*p* = 0.052), pleasantness toward drinking water (*p* = 0.211), dryness in the mouth (*p* = 0.072), taste in the mouth (*p* = 0.12), fullness (*p* = 0.099), and sickness (*p* = 0.145) between trial, recovery condition, and time; however, it can be observed that feelings of thirstiness and dryness in the mouth trended toward significance in this three-way interaction. Despite no significant three-way interactions, there were significant trial × time interactions for thirstiness (*p* = 0.004), pleasantness in the mouth (*p* = 0.034), dryness in the mouth (*p* = 0.002), and fullness (*p* = 0.034). Specifically, thirstiness was significantly greater at POST_EX_ (ES = 5.81) and POST_REC_ (ES = 3.48) in the HYPO trial (POST_EX_, 69.5 ± 9.0; POST_REC_, 51.9 ± 14.1) than EUH trial (POST_EX_, 23.1 ± 6.8; POST_REC_, 15.5 ± 4.4). Pleasantness in the mouth was significantly greater (greater unpleasant feeling) at POST_EX_ (ES = 8.01) and POST_REC_ (ES = 3.95) in the HYPO trial (POST_EX_, 84.9 ± 5.9; POST_REC_, 67.3 ± 10.6) than in the EUH trial (POST_EX_, 36.4 ± 6.2; POST_REC_, 32.1 ± 6.8). Dryness in the mouth was significantly greater at POST_EX_ (ES = 6.48) and POST_REC_ (ES = 3.18) in the HYPO trial (POST_EX_, 70.3 ± 5.9; POST_REC_, 60.6 ± 12.3) than in the EUH trial (POST_EX_, 26.2 ± 7.6; POST_REC_, 29.2 ± 6.6). Lastly, participants experienced significantly greater fullness at POST_EX_ (ES = 2.93) in the EUH trial (39.5 ± 9.0) compared to the HYPO trial (16.9 ± 6.1).

**Figure 3:**
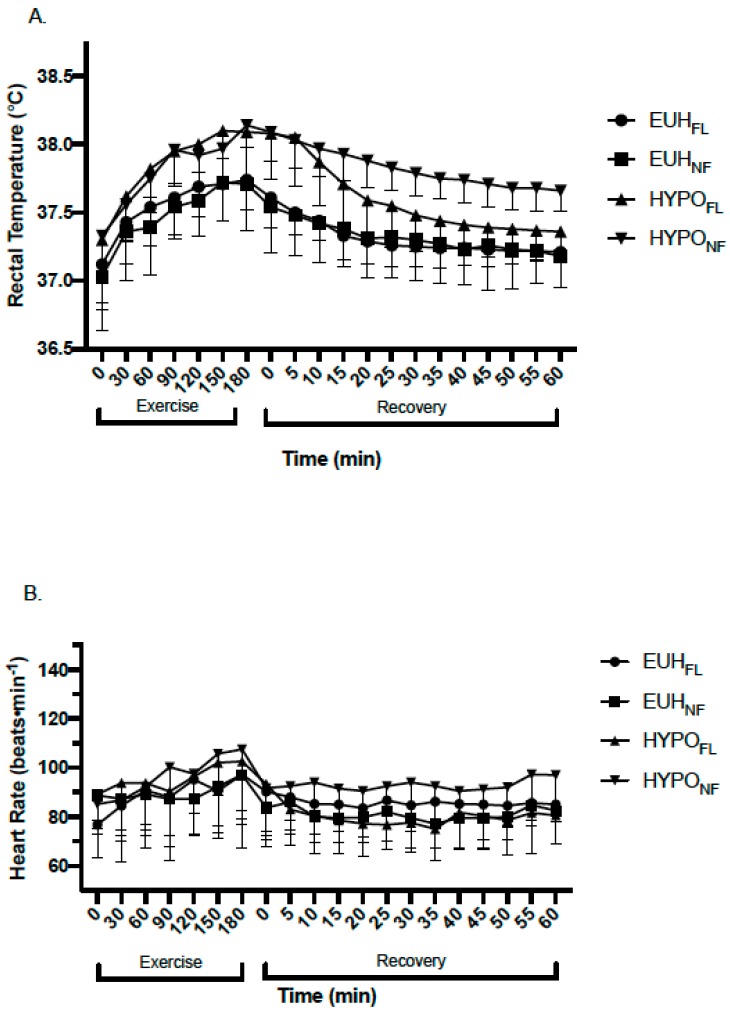
Changes in (**A**) rectal temperature and (**B**) heart rate during exercise and recovery in EUH_FL_, EUH_NF_, HYPO_FL_, and HYPO_NF_ groups. EUH_FL_ = minimized fluid losses during exercise and remaining losses replaced during recovery; EUH_NF_ = minimized fluid losses during exercise and did not replace losses during recovery; HYPO_FL_ = fluid restricted during exercise and losses replaced during recovery; HYPO_NF_ = fluid restricted during exercise and losses not replaced during recovery.

The hypohydrated trials (HYPO_FL_ and HYPO_NF_) resulted in significantly greater levels of dehydration at POST_EX_ and POST_REC_ than the euhydrated trials (EUH_FL_ and EUH_NF_), as measured by the percentage of body mass loss (%BML) (*p* < 0.001) (Table 1). Fluid replacement after exercise did not influence %BML between HYPO trials (HYPO_FL_, 2.1% ± 1.1%; HYPO_NF_, 2.6% ± 0.6%) or EUH trials (EUH_FL_, 0.2% ± 0.7%; EUH_NF_, 0.6% ± 0.5%) at POST_REC_, respectively (*p* = 0.330) (Table 1). Serological and urinary hydration measures are shown in Table 2. Changes in T_REC_ (*p* = 0.052) and HR (*p* = 0.067) trended toward statistical significance (Figure 3). 

## 4. Discussion

This study evaluated the use of thirst as a marker of hydration following exercise-induced dehydration. Subjective sensations of thirst (TH and TSS) were significantly elevated immediately following an exercise bout that induced a level of hypohydration of 2.8% ± 0.9% body mass loss (combined mean of HYPO_FL_ and HYPO_NF_ groups). Our hypothesis was supported in that when HYPO_FL_ was permitted to consume water ad libitum during the initial 10 min of a 60 min bout of recovery following exercise, subjective feelings of thirst (TH) were minimized throughout and at the completion of recovery to levels similar to the EUH_FL_ and EUH_NF_ groups, despite %BML remaining >2% (Figure 1A). Measures of thirstiness and dryness in the mouth, as measured by TSS, approached statistical significance (*p* = 0.052 and *p* = 0.072, respectively), with HYPO_FL_ exhibiting a reduction in thirst and dryness in the mouth following 60 min of recovery (Figure 2).

The role of oropharyngeal receptors in attenuating thirst has been extensively studied in both human [11,17,23,24,25,26] and animal models [27,28,29,30]. Figaro and Mack [11] showed that oropharyngeal receptors played an immediate role in inhibiting thirst, and thus, fluid intake, without influencing plasma osmolality when fluid was extracted immediately from the stomach after consumption. In addition, Mears et al. [17] found that thirst, which was stimulated by a rise in serum osmolality following a bout of high intensity interval exercise, rapidly declined upon the consumption of fluid immediately after exercise. Thirst also remained elevated when fluid consumption was delayed for 30 min or prohibited at any point during recovery, despite a decline in serum osmolality. Our findings support these results and the role that oropharyngeal receptors play in reducing the thirst sensation, despite a sustained elevation of serum osmolality, in that the sensation of thirst was immediately reduced in HYPO_FL_ once fluid was permitted and remained at levels similar to EUH_FL_ and EUH_NF_ (Figure 1A and Figure 2), despite a sustained elevation in serum osmolality levels compared to baseline (+14 mOsm·kg^−1^). This is evident in Figure 1B, where the delta change of pooled means for TH between recovery and exercise shows a negative delta change for EUH_FL_, EUH_NF_, and HYPO_FL_ compared to HYPO_NF_; this figure shows that sensations of thirst for the former conditions are lower across the recovery period on average than what appeared during exercise. 

Interestingly, unlike the work by Figaro and Mack [11] and Mears et al. [17], we did not see a decline in serum osmolality when ad libitum fluid intake was permitted. We postulate that this may be due to the total volume of water consumed. In the Figaro and Mack [11] and Mears et al. [17] studies, participants replaced ~67–86% and 63–82% of the water lost during exercise, respectively. In our study, participants in HYPO_FL_ only replaced ~55% of what they lost, which may have influenced the amount of water absorbed into the vasculature during recovery. As we did not measure serum osmolality immediately following exercise or throughout the recovery portion of the trial, nor did we measure the amount of fluid absorbed from the gastrointestinal tract during recovery, we are unable to develop a further rationale as to why we did not observe a change in serum osmolality. We postulate that the total volume of water consumed during the initial 10 min of recovery was not large enough to result in a change in serum osmolality.

The role of one’s mouth’s state may be an important factor when considering fluid replacement following exercise eliciting levels of dehydration that exceed 2% BML, a level of dehydration that has been shown to adversely affect physiological function [31,32,33,34] and exercise performance [4,35,36,37]. Our findings, in support of prior literature [10,21,26], show that individuals will have a reduced drive for consuming fluids once sensations of thirst, dryness in the mouth, and unpleasantness in the mouth are rectified and prior to completing fluid replacement; this incomplete fluid replacement has commonly been termed “involuntary” or “voluntary” dehydration [6,38]. Specifically, in our study, thirst, mouth dryness, and ratings of unpleasantness in the mouth in HYPO_FL_ were lower at POST_REC_ than POST_EX_ (Figure 3), despite a level of hypohydration of 2.1% ± 1.1%, a level of body mass loss where thirst is typically induced [6,9]. Despite not being statistically significant, thirst (*p* = 0.052) and dryness in the mouth (*p* = 0.072) exhibited a large effect for HYPO_NF_ at POST_REC_ when compared to HYPO_FL_ (ES = 7.15 and 6.3, respectively), EUH_FL_ (ES = 7.49 and 6.02, respectively), and EUH_NF_ (ES = 5.6 and 5.61, respectively) conditions. 

We observed that within the first 10 min of recovery, participants in HYPO_FL_ consumed approximately 55% of total fluid losses incurred during exercise, which is consistent with prior literature [11,17]. It must be noted, however, that we only permitted participants 10 min to consume water, which may have prevented additional consumption to offset fluid losses. Interestingly, Evans et al. [39] assessed ad libitum intake of fluids at 15 min increments for 2 h following exercise; their findings show that after 15 min of ad libitum fluid consumption, roughly 25–30% of fluid losses were replaced. Speculating as to the reason for this discrepancy, knowledge from the participants of how long they had access to fluid in the current study may have prompted them to consume more fluid than they would have if allotted more time overall to consume fluids. Despite this, we believe that if our participants were permitted to consume water throughout the entire 60 min recovery period, they would not fully replace fluid losses. Work by Maughan et al. [40,41,42] and Shirreffs et al. [43,44] suggest that following exercise, especially when there is limited time before the next bout, a strategic approach to rapid rehydration based on individual losses must be utilized to optimize the potential for rehydration. Relying solely on thirst alone would not be appropriate in this scenario, especially if fluid losses exceed ~3% of body mass, as shown in our study.

To contextualize the aforementioned into real-world context, allowing participants to consume fluids during the first 10 min of a post-exercise recovery period, may mimic what could occur in a sport. For example, sports such as soccer and rugby, require athletes to perform continuous exercise, with the elite levels of these sports preventing the number of substitutions permitted; this could create a scenario in which athletes enter the half-time portion of a competition (typically 10–15 min in length) hypohydrated to ~2% BML, especially if competition is being performed in hot conditions. If provided ad libitum access to fluids, based on our findings, these athletes would consume fluids to quench their thirst, and if only using thirst as a measure of preparedness for the second half of competition, would enter the latter half of the event hypohydrated to a level that may result in marked performance deficits. While our study did not examine whether thirst would remain attenuated if a second bout of exercise ensued based on the aforementioned example, our findings would support the recommendation that individualized fluid replacement strategies are optimal for minimizing the extent of fluid losses during exercise.

This study is not without limitations. We only tested male participants in this study, which may not be generalizable to females, particularly given the physiological changes that occur during the menstrual cycle that may influence hydration state and thirst [45]. Furthermore, we only permitted participants to consume water ad libitum for a 10 min block of time immediately post exercise. Without permitting ad libitum consumption of water for the entire duration of post-exercise recovery, we were unable to determine if fluid consumption would have continued to further correct fluid losses. Additionally, given the influence of increasing the osmolarity of the fluid that is being consumed and the attenuation in the decline of thirst, we are unable to make a determination of how the osmolarity of fluid following exercise-induced hypohydration may have further augmented the replacement of fluids. Evans et al. [39] found no difference of ad libitum fluid ingested when comparing hypertonic 10%, 2%, and 0% glucose solutions; however, since an equal concentration of sodium was included in each beverage, it is unknown if differences would have been found if plain water was also ingested. Furthermore, the thirst scales utilized, TH [20] and TSS [21,22], have not been validated to date; there is no existing evidence that has compared changes in plasma osmolality to the thirst scales utilized for this study. While this prevents us from making conclusions based on perceptual scales validated against physiological measures, in utilizing a randomized cross-over design where participants completed both a euhydrated and hypohydrated trial under the same environmental conditions and exercise stress, we feel that the within-person changes in the thirst scales tested allows for consistency in these measures. Our post hoc power analysis revealed that we were underpowered for the TSS measures, which may explain why we observed non-significant findings for thirstiness, dryness in the mouth, pleasantness in the mouth, and fullness. Lastly, by not utilizing an exercise duration and/or intensity that may mimic various settings (athletic, occupational, and military settings) we are not able to make conclusive statements surrounding the use of thirst in guiding fluid replacement following the cessation of exercise. 

## 5. Conclusions

In conclusion, our findings indicate that when a bolus of fluid is provided immediately following exercise-induced dehydration, the sensation of thirst rapidly declines to levels observed in euhydrated individuals for up to 60 min following exercise, despite a level of dehydration exceeding 2% body mass loss. The prolonged inhibition of thirst when less fluid was consumed than total water losses may prevent one’s ability to rehydrate rapidly following prolonged exercise. These findings support the recommendation that individuals may benefit from knowing their fluid needs and that fluid replacement should be individualized based on fluid losses and subsequent fluid need. Future research should consider examining the types of fluids consumed and allowing participants consume fluids ad libitum at their discretion following exercise-induced dehydration. This would further expand on the utility of thirst as a tool to guide fluid replacement following exercise-induced dehydration and provide for more refined, data-informed recommendations being derived.

## Figures and Tables

**Table 1:** Body mass changes by condition.

Condition	PRE_EX_ Mass (kg)	POST_EX_ Mass (kg)	POST_EX_ %BML (%)	POST_REC_ Mass (kg)	POST_REC_ %BML (%)	POST_REC_ Fluid Consumed (mL)	% BML Replaced (%)
EUH_FL_	77.0 ± 10.9	76.8 ± 10.2	0.2 ± 0.7	76.9 ± 11.1	0.2 ± 0.7	337.5 ± 48.3	57.4 ± 13.2
EUH_NF_	85.9 ± 6.3	85.4 ± 6.7	0.6 ± 0.7	85.4 ± 6.3	0.6 ± 0.5	0 ± 0	0 ± 0
HYPO_FL_	76.4 ± 11.3	74.1 ± 11.0	3.0 ± 1.2 ^α,β^	74.8 ± 11.4	2.1 ± 1.1 ^α,β^	1100.0 ± 155.0	55.3 ± 15.6
HYPO_NF_	86.4 ± 6.5	84.1 ± 6.1	2.6 ± 0.6 ^χ,δ^	84.1 ± 6.5	2.6 ± 0.6 ^χ,δ^	0 ± 0	0 ± 0

α = HYPO_FL_ significantly different than EUH_FL_, *p* < 0.05; β = HYPO_FL_ significantly different than EUH_NF_, *p* < 0.05; χ = HYPO_NF_ significantly different than EUH_FL_, *p* < 0.05; δ = HYPO_NF_ significantly different than EUH_NF_, *p* < 0.05.

**Table 2:** Serological and urinary hydration variables.

Variable	Condition	PRE_EX_	POST_REC_
Serum Osmolality (mOsm·kg^−1^)	EUH_FL_	295 ± 2	296 ± 3
	EUH_NF_	290 ± 5	291 ± 5
	HYPO_FL_	296 ± 8	310 ± 6 ^β,^*
	HYPO_NF_	294 ± 3	304 ± 3 ^δ,^*
Urine Specific Gravity (AU)	EUH_FL_	1.012 ± 0.009	1.009 ± 0.004
	EUH_NF_	1.018 ± 0.005	1.017 ± 0.007
	HYPO_FL_	1.014 ± 0.008	1.017 ± 0.004
	HYPO_NF_	1.021 ± 0.007	1.020 ± 0.005

PRE_EX_, prior to commencement of exercise; POST_REC_, 60 min post exercise. β = HYPO_FL_ significantly different than EUH_NF_, *p* < 0.05; δ = HYPO_NF_ significantly different than EUH_NF_, *p* < 0.05; * = POST significantly greater than PRE, *p* < 0.05.

## References

[1] Armstrong L.E. (2005). Hydration assessment techniques. Nutr. Rev..

[2] Armstrong L.E. (2007). Assessing Hydration Status: The Elusive Gold Standard. J. Am. Coll. Nutr..

[3] Cheuvront S.N., Ely B.R., Kenefick R.W., Sawka M.N. (2010). Biological variation and diagnostic accuracy of dehydration assessment markers. Am. J. Clin. Nutr..

[4] Cheuvront S.N., Kenefick R.W. (2014). Dehydration: Physiology, Assessment, and Performance Effects. Compr. Physiol..

[5] Cheuvront S.N., Kenefick R.W., Charkoudian N., Sawka M.N. (2013). Physiologic basis for understanding quantitative dehydration assessment. Am. J. Clin. Nutr..

[6] Greenleaf J.E. (1992). Problem: Thirst, drinking behavior, and involuntary dehydration. Med. Sci. Sports Exerc..

[7] McKinley M.J., Johnson A.K. (2004). The Physiological Regulation of Thirst and Fluid Intake. Physiology.

[8] Stanhewicz A.E., Kenney W.L. (2015). Determinants of water and sodium intake and output. Nutr. Rev..

[9] Wolf A. (1950). Thirst; Physiology of the Urge to Drink and Problems of Water Lack.

[10] Brunstrom J.M., Tribbeck P.M., Macrae A.W. (2000). The role of mouth state in the termination of drinking behavior in humans. Physiol. Behav..

[11] Figaro M.K., Mack G.W. (1997). Regulation of fluid intake in dehydrated humans: Role of oropharyngeal stimulation. Am. J. Physiol. Integr. Comp. Physiol..

[12] Seckl J.R., Williams T.D.M., Lightman S.L. (1986). Oral hypertonic saline causes transient fall of vasopressin in humans. Am. J. Physiol..

[13] Hew-Butler T., Rosner M.H., Fowkes-Godek S., Dugas J.P., Hoffman M.D., Lewis D.P., Maughan R.J., Miller K.C., Montain S.J., Rehrer N.J. (2015). Statement of the 3rd International Exercise-Associated Hyponatremia Consensus Development Conference, Carlsbad, California, 2015. Br. J. Sports Med..

[14] Sawka M.N., Burke L.M., Eichner E.R., Maughan R.J., Montain S.J., Stachenfeld N.S. (2007). American College of Sports Medicine position stand. Exercise and fluid replacement. Med. Sci. Sports Exerc..

[15] McDermott B.P., Anderson S.A., Armstrong L.E., Casa D.J., Cheuvront S.N., Cooper L., Kenney W.L., O’Connor F.G., Roberts W.O. (2017). National Athletic Trainers’ Association Position Statement: Fluid Replacement for the Physically Active. J. Athl. Train..

[16] Armstrong L.E., Ganio M.S., Klau J.F., Johnson E.C., Casa D.J., Maresh C.M. (2014). Novel hydration assessment techniques employing thirst and a water intake challenge in healthy men. Appl. Physiol. Nutr. Metab..

[17] Mears S.A., Watson P., Shirreffs S.M. (2016). Thirst responses following high intensity intermittent exercise when access to ad libitum water intake was permitted, not permitted or delayed. Physiol. Behav..

[18] Armstrong L.E., Maresh C.M., Castellani J.W., Bergeron M.F., Kenefick R.W., Lagasse K.E., Riebe D. (1994). Urinary Indices of Hydration Status. Int. J. Sport Nutr..

[19] Jackson A.S., Pollock M.L. (1978). Generalized equations for predicting body density of men. Br. J. Nutr..

[20] Engell D.B., Maller O., Sawka M.N., Francesconi R.N., Drolet L., Young A.J. (1987). Thirst and fluid intake following graded hypohydration levels in humans. Physiol. Behav..

[21] Rolls B.J., Wood R.J., Rolls E.T., Lind H., Lind W., Ledingham J.G. (1980). Thirst following water deprivation in humans. Am. J. Physiol. Integr. Comp. Physiol..

[22] Phillips P.A., Rolls B.J., Ledingham J.G.G., Forsling M.L., Morton J.J., Crow M.J., Wollner L. (1984). Reduced thirst after water deprivation in healthy elderly men. N. Engl. J. Med..

[23] Salata R.A., Verbalis J.G., Robinson A.G. (1987). Cold Water Stimulation of Oropharyngeal Receptors in Man Inhibits Release of Vasopressin. J. Clin. Endocrinol. Metab..

[24] O’Obika L.F., O’Okpere S., O’Ozoene J., Amabebe E. (2014). The role of oropharnygeal receptors in thirst perception after dehydration and rehydration. Niger. J. Physiol. Sci..

[25] Arnaoutis G., Kavouras S.A., Christaki I., Sidossis L.S. (2012). Water Ingestion Improves Performance Compared with Mouth Rinse in Dehydrated Subjects. Med. Sci. Sports Exerc..

[26] Phillips P.A., Rolls B.J., Ledingham J.G., Morton J.J. (1984). Body fluid changes, thirst and drinking in man during free access to water. Physiol. Behav..

[27] Thrasher T.N., Nistal-Herrera J.F., Keil L.C., Ramsay D.J. (1981). Satiety and inhibition of vasopressin secretion after drinking in dehydrated dogs. Am. J. Physiol. Metab..

[28] Appelgren B.H., Thrasher T.N., Keil L.C., Ramsay D.J. (1991). Mechanism of drinking-induced inhibition of vasopressin secretion in dehydrated dogs. Am. J. Physiol. Integr. Comp. Physiol..

[29] Thornton S.N., Baldwin B.A., Forsling M.L. (1989). Drinking and vasopressin release following central injections of angiotensin II in minipigs. Q. J. Exp. Physiol. Transl. Integr..

[30] Blair-West J.R., Gibson A.P., Woods R.L., Brook A.H. (1985). Acute reduction of plasma vasopressin levels by rehydration in sheep. Am. J. Physiol..

[31] Sawka M.N., Young A.J., Francesconi R.P., Muza S.R., Pandolf K.B. (1985). Thermoregulatory and blood responses during exercise at graded hypohydration levels. J. Appl. Physiol..

[32] Montain S.J., Coyle E.F. (1992). Influence of graded dehydration on hyperthermia and cardiovascular drift during exercise. J. Appl. Physiol..

[33] Adams W.M., Mazerolle S.M., Casa D.J., Huggins R.A., Burton L. (2014). The Secondary School Football Coach’s Relationship with the Athletic Trainer and Perspectives on Exertional Heat Stroke. J. Athl. Train..

[34] Huggins R., Martschinske J., Applegate K., Armstrong L., Casa D. (2012). Influence of Dehydration on Internal Body Temperature Changes During Exercise in the Heat: A Meta-Analysis. Med. Sci. Sports Exerc..

[35] Casa D.J., Stearns R.L., Lopez R.M., Ganio M.S., McDermott B.P., Yeargin S.W., Yamamoto L.M., Mazerolle S.M., Roti M.W., Armstrong L.E. (2010). Influence of Hydration on Physiological Function and Performance During Trail Running in the Heat. J. Athl. Train..

[36] Adams J.D., Sekiguchi Y., Suh H.G., Seal A.D., Sprong C.A., Kirkland T.W., Kavouras S.A. (2018). Dehydration Impairs Cycling Performance, Independently of Thirst: A Blinded Study. Med. Sci. Sports Exerc..

[37] Armstrong L.E., Costill D.L., Fink W.J. (1985). Influence of diuretic-induced dehydration on competitive running performance. Med. Sci. Sports Exerc..

[38] Greenleaf J.E., Sargent F. (1965). Voluntary dehydration in man. J. Appl. Physiol..

[39] Evans G.H., Shirreffs S.M., Maughan R.J. (2009). Postexercise rehydration in man: The effects of carbohydrate content and osmolality of drinks ingested ad libitum. Appl. Physiol. Nutr. Metab..

[40] Maughan R., Lamb D., Gisolfi C. (1997). Optimizing Hydration for Competitive Sport. Perspectives in Exercise Science and Sport Medicine.

[41] Maughan R.J., Shirreffs S.M. (1998). Dehydration, rehydration and exercise in the heat: Concluding remarks. Int. J. Sports Med..

[42] Maughan R.J., Shirreffs S.M. (2010). Dehydration and rehydration in competative sport. Scand. J. Med. Sci. Sports.

[43] Shirreffs S.M. (2001). Restoration of fluid and electrolyte balance after exercise. Can. J. Appl. Physiol..

[44] Shirreffs S.M., Maughan R.J. (1998). Volume repletion after exercise-induced volume depletion in humans: Replacement of water and sodium losses. Am. J. Physiol. Physiol..

[45] Robertson G. (1984). Abnormalities of thirst regulation. Kidney Int..

